# Lessons from the pandemic: Relationship between patient visits and patient length of stay in California’s health system

**DOI:** 10.1186/s12961-024-01283-8

**Published:** 2025-01-21

**Authors:** David D. Cho, Yu Wang

**Affiliations:** 1 Department of Management, College of Business and Economics, California State University, Fullerton, Fullerton, CA 92831 United States of America; 2 Congdon School of Supply Chain, Business Analytics, and Information Systems, University of North Carolina Wilmington, Wilmington, NC 28403 United States of America

**Keywords:** Health care utilization, Average patient length of stay, Quality of care, California hospitals, COVID-19 pandemic

## Abstract

**Background:**

The coronavirus disease 2019 (COVID-19) pandemic placed a heavy strain on the United States healthcare system. Common hospital operational performances were impacted to varying degrees by the pandemic. This study examined the healthcare operational measures during COVID-19 pandemic.

**Methods:**

This cross-sectional study examined the COVID-19 cases and death counts of 56 California counties and hospital-level utilization data of 397 hospitals in California from January 2019 to March 2021.

**Results:**

A total of 56 California counties were analysed, of which 37 counties were urban and 19 counties were rural. Average patient length of stay was positively associated with the number of intensive care unit visits by COVID-19 patients for all counties, as well as urban and rural counties separately. However, average patient length of stay was negatively associated with the number of inpatient visits by COVID-19 patients for all counties and urban counties.

**Conclusions:**

The findings suggest that, while the need for additional beds and nursing staff in intensive care units exceeded initial estimates, there were also opportunities to streamline the care process for improved efficiency in regular acute care units. The understanding of factors impacting average patient length of stay would be valuable for hospital administrators in optimizing resource allocation and utilization to balance patient outcomes with financial sustainability during disruptive events.

## Background

The COVID-19 pandemic has brought about profound and far-reaching effects on the United States healthcare system. Since its initial outbreak, researchers have identified and studied the impact of COVID-19 in many areas. As the virus spread rapidly across the United States in the early stages, many hospitals and health care facilities became overwhelmed with surges of patients seeking care. This increase in demand placed a heavy strain on health care workers, leading to fatigue, burnout and staffing shortages [1–3]. To prioritize COVID-19 patients and conserve resources, hospitals and health care facilities frequently had to postpone or cancel routine and elective procedures such as surgeries, screenings and other nonurgent medical care [4, 5]. This disruption led to delays in the diagnosis and treatment of health conditions unrelated to COVID-19, which could have long-term implications for patients’ health outcomes [6]. Since routine and elective procedures are also important contributors to a hospital’s financial health, accounting for approximately 63% of an average hospital’s revenue in 2018 [7], their disruption also posed serious challenges to the financial solvency of many hospitals [8, 9]. Researchers have conducted various COVID-19-related difference-in-differences studies such as urban vs rural [10], safety-net vs non-safety-net hospitals [11] and vaccinated vs unvaccinated outcomes [12, 13]. In this study, we contribute to the investigation of COVID-19’s impact on the United States health care system by looking at operational performance outcomes. More specifically, we are interested in studying how various operational measures, such as daily hospital visits, revenue per discharge and average patient length of stay (ALOS), evolved from the initial outbreak to the arrival of the first vaccine.

During the peak of the COVID-19 pandemic, hospitals faced overwhelming demand from both regular and COVID-19-related patient arrivals, forcing many health care organizations to operate beyond capacity for extended periods. This has contributed to significant stress and increased burnout among staff members, potentially impacting the quality of care [14]. Therefore, it is reasonable to hypothesize that the common patient performance measures, such as ALOS, may be negatively associated with COVID-19 patient arrivals. To accommodate the surge in demand, many hospitals called for additional resources and extra staff [14, 15], and in many cases, hospitals prioritized COVID-19 patients – who presented varying levels of acuity and severity – over nonurgent cases. This, however, introduced complexities, making the actual impact on ALOS more nuanced and difficult to evaluate.

ALOS is a critical metric widely used in healthcare research to measure the duration of time a patient spends in a health care system, from admission to discharge. It is widely used to provide insights into resource utilization [16, 17], quality of care [18, 19], patient outcomes [20, 21] and health policy development [22], making it particularly relevant for researchers, health care providers, policymakers and other stakeholders. For hospital administrators, ALOS directly informs capacity management, resource allocation and staffing decisions, particularly during periods of high patient demand [23, 24]. Policymakers, including those overseeing Medicare and Medicaid, use ALOS as a key performance measure to assess hospital efficiency and determine reimbursement levels. Under bundled payment models, shorter ALOS is incentivized to improve patient throughput and reduce costs, creating financial motivations to optimize care delivery without compromising quality [25]. By identifying trends in ALOS, stakeholders can develop targeted strategies to improve the effectiveness of health care delivery, leading to better quality of patient care.

This investigation utilizes data from 56 California counties spanning the period from 2019 quarter 1 (Q1) to 2021 Q1. Three primary research questions guide our study. First, how did hospital operational characteristics change after the onset of the COVID-19 pandemic? Second, what is the association between patient average length of stay and the number of COVID-19 hospital visits? Lastly, are the outcomes divergent between rural and urban counties concerning the first two research questions?

## Methods

### Study design

We conducted a cross-sectional study encompassing 56 out of all 58 California counties over a span of nine quarters (2019 Q1 to 2021 Q1), capturing both a complete pre-pandemic calendar year and the initial five quarters of the COVID-19 pandemic. We focused on periods before the wide availability of COVID-19 vaccine [26] and at-home rapid tests [27], since vaccines and rapid tests are shown to be associated with underreporting of COVID-19 cases [28]. This may, in turn, compromise the validity of performance measures such as infection rate after 2021 Q1. Two counties, Alpine (population: 1235) and Sierra (population: 3283), were excluded from the analysis owing to missing hospital-level utilization data. Moreover, noncomparable hospitals categorized under psychiatric hospitals and long-term care hospitals were omitted from hospital-level data to focus on general hospitals. This study used publicly available county-level and hospital-level data and was determined to be exempt from the informed consent requirement by the institutional review board.

### Data

This study curated a consolidated dataset from a synthesis of five distinct sources. We obtained county-level COVID-19 total cases and death counts from the Statewide COVID-19 Cases Deaths Tests dataset [29], and hospitalized cases and deaths data from the Statewide COVID-19 Hospital County Data [30]. Both datasets are available on the California Open Data Portal and published by the California Department of Public Health. The demographic characteristics of California counties were sourced from the United States Census Bureau [31]. Hospital-level utilization data were extracted from the Hospital Quarterly Financial and Utilization Data from the State of California Department of Health Care Access and Information [32]. To align the data, we transformed hospital-level operational measures into county-level data by aggregating the measures for each county on the basis of the physical locations of health care facilities. The rural and urban designations for the counties were determined on the basis of the designations provided by the State of California Department of Justice, adhering to the definitions outlined by the United States Department of Agriculture [33].

### Statistical analysis

We examined the impact of the COVID-19 pandemic on various county-level operational metrics, including infection rate, death rate, number of beds per capita, revenue per discharge and revenue per day. Infection and death rates were calculated by dividing the number of COVID-19 cases and related deaths by the population of each county in the state. To determine the number of beds per capita, the available beds across all hospitals in a county were divided by the county’s population. Revenue per discharge and per day for each county were computed by dividing the gross inpatient revenue by the total number of discharges and total patient days, respectively, following the definitions and formulas provided by the California Department of Health Care Access [34]. Additionally, following the rural and urban classifications provided by the State of California Department of Justice, the average value of each variable was calculated for overall, rural and urban counties.

We conducted fixed-effects panel regression analysis to evaluate the association between ALOS and the county-level operational metrics. ALOS, the outcome variable, was calculated by dividing the total patient days by the total hospital discharges in each county, adhering to the definition and formula provided by the California Department of Health Care Access and Information (formerly the Office of Statewide Health Planning and Development) [34]. The independent variables were the number of inpatient visits by COVID-19 patients, the number of intensive care unit (ICU) visits by COVID-19 patients, infection rate, death rate, number of beds per capita and revenue per discharge. For both inpatient and ICU visits, we aggregated the total visits by COVID-19 patients across all hospitals within a county for each quarter. Subsequently, the average daily visits for each quarter were calculated by dividing the aggregated figure by the number of days in that quarter. The regression model included quarter–year dummy variables and county fixed effects to account for unobserved heterogeneity across counties. Standard errors were clustered at the county level to account for within-cluster correlation and heteroskedasticity.

This cross-sectional study followed the Strengthening the Reporting of Observational Studies in Epidemiology (STROBE) reporting guideline. When statistical tests were performed, statistical significance was set at two-sided $P<0.05$. Statistical analyses were performed using STATA 16.1 (STATA Corp, College Station, TX).

## Results

### Counties and hospital operational characteristics

Table 1 illustrates the aggregated operational characteristics of counties prior to and following the onset of the pandemic. The average availability of beds per 1000 population showed a slight decline from 2.29 to 2.27 across all counties during the pandemic. However, it is worth noting that urban counties experienced a decrease, with available beds per 1000 population dropping from 2.30 to 2.28. In contrast, rural counties exhibited an increase, with available beds per 1000 population rising from 1.75 to 1.77. Regarding revenue per discharge, both urban and rural counties observed an upwards trend during the pandemic. Urban counties witnessed an increase from US$ 84,443 (standard deviation: US$ 28,181) to US$ 101,859 (standard deviation: US$ 33,919), while rural counties experienced a rise from US$ 50,288 (standard deviation: US$ 17,840) to US$ 60,029 (standard deviation: US$ 29,136). This increase in revenue per discharge is consistent with findings in the literature: the interventions of government assistance programs were crucial in maintaining the financial solvency of many health care organizations [11]. Policies such as the 20% Medicare payment increase for inpatient COVID-19 admissions helped hospitals maintain or boost revenue per discharge. Additionally, relief initiatives such as the Provider Relief Fund (PRF) and the Paycheck Protection Program helped many hospitals control their operating expenses. Consequently, many hospitals were able to survive financially during the pandemic, and some remained profitable even at its peak [11, 35].

**Table 1:** County and hospital characteristics by urban–rural classifications

Characteristics	Mean		
	All (*n* = 56)	Urban (*n* = 37)	Rural (*n* = 19)
Total population	701,418	1,039,182	43,668
Available beds, pre-pandemic	1604	2388	76
Available beds, post-pandemic	1595	2366	78
Available beds per 1000 population, pre-pandemic	2.29	2.30	1.75
Available beds per 1000 population, post-pandemic	2.27	2.28	1.77
Revenue per discharge, pre-pandemic, in US$	72,855	84,443	50,288
Revenue per discharge, post-pandemic, in US$	87,766	101,859	60,029

Data are the United States Census Bureau and the Hospital Quarterly Financial and Utilization Data from the State of California Department of Health Care Access and Information. Pre-pandemic periods are from 2019 Q1 to 2019 Q4. Post-pandemic periods are from 2020 Q1 to 2021 Q1. The sample contains 397 hospitals across 56 counties.

**Fig. 1:**
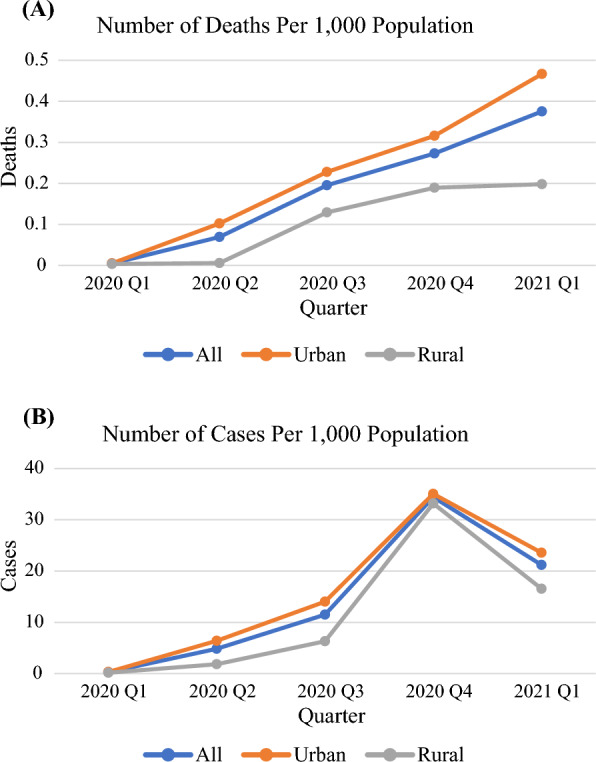
Number of confirmed COVID-19 associated deaths (**A**) and cases (**B**) per 1000 population from 2020 Q1 to 2021 Q1

**Fig. 2:**
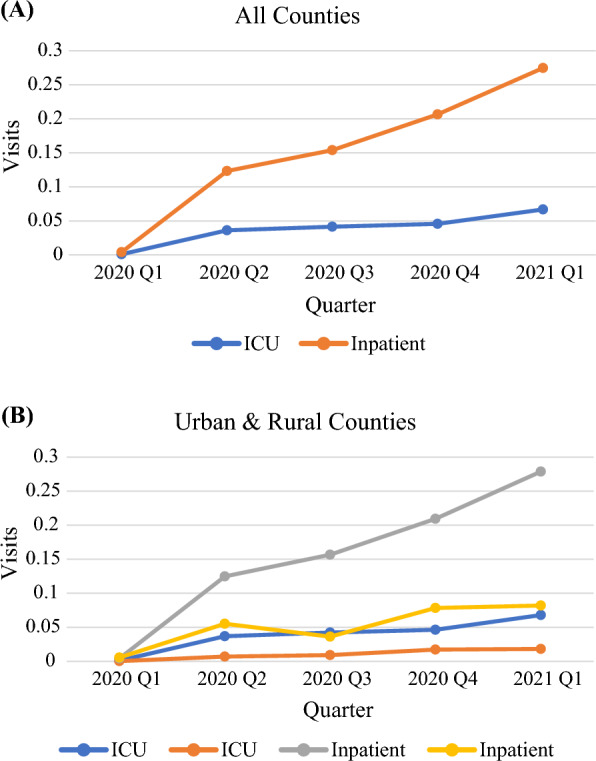
Average daily visits by COVID-19 patients per 1000 population for all counties (**A**) and by urban-rural classifications (**B**) from 2020 Q1 to 2021 Q1

The COVID-19-related death rate and infection rate were evaluated starting 2020 Q1. The average COVID-19-related death rate increased every quarter from 2020 Q1 to 2021 Q1 for all counties, but urban counties experienced a steeper increase. The notable increase in death rate also did not start until 2020 Q2 for rural counties (Fig. 1A). The COVID-19 infection rate steadily increased for the duration of our data and peaked in 2020 Q4. Afterwards, a sharp decline was observed in 2021 Q1, which coincides with the introduction of vaccines (Fig. 1B). The daily average number of COVID-19 ICU and inpatient visits per 1000 population increased every quarter for the state overall (Fig. 2). The daily average number of inpatient visits per 1000 population decreased for rural counties (from 0.055 to 0.036) in 2020 Q3, but it increased again in both 2020 Q4 and 2021 Q1.

### Average patient length of stay

**Fig. 3:**
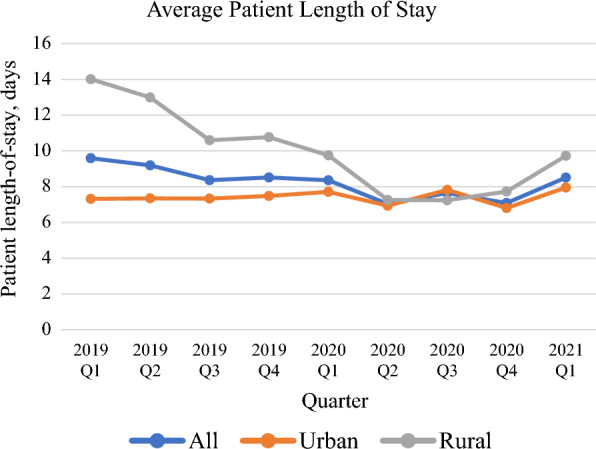
Average patient length of stay from 2019 Q1 to 2021 Q1

The overall ALOS decreased every quarter before the onset of the pandemic, except for 2019 Q4 (Fig. 3). After the onset of the pandemic, ALOS decreased considerably from 2020 Q1 to 2020 Q2 (from 8.35 to 7.03 days). While it stayed less than 8 days, it increased again to greater than 8 days in 2021 Q1.

When all counties were considered, our regression analysis results showed a significant association between ALOS and both the number of inpatient visits by COVID-19 patients and the number of ICU visits by COVID-19 patients (Table 2). While the association was negative between ALOS and the number of inpatient visits by COVID-19 patients (coefficient: −0.00030, 95% confidence interval (CI) −0.00055 to −0.000047, *P* = 0.021), it was positive between ALOS and the number of ICU visits by COVID-19 patients (coefficient: 0.0012, 95% CI 0.00018 to 0.0022, *P* = 0.021). A significant and positive association was also observed between ALOS and average revenue per patient discharge (coefficient: 0.45, 99.9% CI 0.22–0.67, *P* = 0). Using 2019 Q1 as a benchmark, we observed statistically significant differences in ALOS from 2020 Q2 to 2021 Q1, but not from 2019 Q2 to 2020 Q1.

**Table 2:** Fixed effects panel regression of average patient length of stay for California counties

Variable	All (*n* = 484)	*P* value	Rural (*n* = 153)	*P* value	Urban (*n* = 331)	*P* value
	Regression coefficient (95% CI)		Regression coefficient (95% CI)		Regression coefficient (95% CI)	
Constant	−6.264 (−12.112 to −0.417)	0.036	−9.201 (−16.428 to −1.975)	0.015	−0.314 (−2.103 to 1.474)	0.724
Number of inpatient visits by COVID-19 patients	−0.00030 (−0.001 to 0)	0.021	−0.0044 (−0.018 to 0.009)	0.512	−0.00027 (0 to 0)	0.016
Number of ICU visits by COVID-19 patients	0.0012 (0 to 0.002)	0.021	0.118 (0.041 to 0.196)	0.005	0.0011 (0 to 0.002)	0.014
Number of beds per capita, log-transformed	−0.491 (−1.23 to 0.248)	0.188	−0.573 (−1.469 to 0.323)	0.196	−0.078 (−0.221 to 0.065)	0.275
Infection rate	0.007 (−0.008 to 0.022)	0.336	0.005 (−0.009 to 0.019)	0.481	0.016 (−0.012 to 0.045)	0.254
Death rate	1.36 (−0.112 to 2.833)	0.070	−2.984 (−6.218 to 0.25)	0.068	1.81 (0.375 to 3.245)	0.015
Revenue per discharge, log-transformed	0.448 (0.224 to 0.673)	0.000	0.686 (0.355 to 1.017)	0	0.142 (0.021 to 0.263)	0.022
2019 Q1	NA, Benchmark Quarter	NA	NA, Benchmark Quarter	NA	NA, Benchmark Quarter	NA
2019 Q2	−0.019 (−0.046 to 0.009)	0.185	−0.032 (−0.09 to 0.026)	0.261	−0.01 (−0.022 to 0.003)	0.130
2019 Q3	−0.035 (−0.075 to 0.004)	0.080	−0.047 (−0.147 to 0.054)	0.341	−0.026 (−0.043 to −0.009)	0.003
2019 Q4	−0.031 (−0.077 to 0.014)	0.170	−0.052 (−0.167 to 0.063)	0.358	−0.018 (−0.044 to 0.008)	0.160
2020 Q1	−0.051 (−0.107 to 0.005)	0.075	−0.082 (−0.207 to 0.043)	0.184	−0.005 (−0.036 to 0.025)	0.731
2020 Q2	−0.071 (−0.129 to −0.013)	0.018	−0.132 (−0.269 to 0.004)	0.057	−0.015 (−0.072 to 0.042)	0.602
2020 Q3	−0.097 (−0.159 to −0.034)	0.003	−0.138 (−0.254 to −0.022)	0.022	−0.044 (−0.098 to 0.01)	0.110
2020 Q4	−0.088 (−0.166 to −0.009)	0.029	−0.117 (−0.242 to 0.007)	0.062	−0.07 (−0.162 to 0.022)	0.131
2021 Q1	−0.108 (−0.184 to −0.032)	0.006	−0.169 (−0.294 to −0.044)	0.011	−0.058 (−0.133 to 0.017)	0.126
Within ${R}^{2}$, %	39.91	NA	54.31	NA	54.44	NA
Between ${R}^{2}$, %	46.21	NA	38.55	NA	31.32	NA

When separating rural and urban counties, the significant and positive association between ALOS and the number of ICU visits by COVID-19 patients was preserved for both rural counties (coefficient: 0.12, 99% CI 0.041–0.20, *P* = 0.005) and urban counties (coefficient: 0.0011, 95% CI 0.00023–0.0019, *P* = 0.014). The significant and negative association between ALOS and the number of inpatient visits by COVID-19 patients was only retained for urban counties (coefficient: −0.00027, 95% CI −0.00049 to −0.000053, *P* = 0.016). The average revenue per patient discharge was once again positively associated with ALOS in both urban (coefficient: 0.14, 95% CI 0.021–0.26, *P* = 0.022) and rural (coefficient: 0.69, 99.9% CI 0.36–1.0, *P* = 0) counties.

## Discussion

In this study, we investigated the changes in common health care operational characteristics in California from 2019 Q1 to 2021 Q1. The decrease in available beds and steeper increase in deaths and cases experienced by urban counties following the onset of the pandemic suggest that the impact of the pandemic was more severe in urban regions. Additionally, the demand surge due to the pandemic was not as high for rural hospitals as for their urban counterparts, and it may have contributed to rural hospitals allowing patients to have longer hospital stays during the pandemic.

The negative association between ALOS and the number of inpatient visits by COVID-19 patients can be attributed to various factors. Hospitals may have premeditatedly implemented procedures to shorten hospital stays, aiming to minimize the risk of COVID-19 transmission within their facilities and to accommodate the increased demand from COVID-19 patients. Additionally, it is possible that the recovery time for most hospitalized COVID-19 patients was generally shorter compared with the average hospital stay from illness unrelated to COVID-19.

Similarly, several factors may have influenced the positive association between ALOS and the number of ICU visits by COVID-19 patients. ICU visits prompted by COVID-19 may have involved more severe cases that required an extended duration of stay. Furthermore, the heightened utilization of ICU units during the pandemic, which has been linked to delays in ICU admissions, may have contributed to the overall lengthening of ALOS [36, 37].

Understanding the association between ALOS and other operational measures can provide valuable insights for hospital administrators and policymakers, particularly in navigating resource allocation and staffing challenges during periods of capacity strain. Our analysis of ALOS can lead to opportunities in optimizing bed allocation, nurse assignments and the employment of temporary staff to improve operational efficiency and care quality. It also validates the prioritization of COVID-19 patients over nonurgent cases during the pandemic, suggesting that this approach may have been even more effective than initially anticipated in managing limited hospital capacity.

While many hospitals turned to agency staff as a solution during peak demand, the prolonged ALOS in ICUs potentially implies an even greater need for additional staffing to sustain care standards. Existing literature suggests that agency staff are typically more expensive and require adequate support to be as effective as regular staff due to unfamiliarity with institutional workflows [38–40]. The higher cost of agency staff can strain hospital budgets, particularly for organizations already operating with thin margins. Their potentially lower efficiency also may impact care delivery, increasing the need for robust oversight and training. On the other hand, owing to shorter ALOS, the large streams of COVID-19 patient arrivals in the regular acute care units represented high patient turnover, which presented opportunities to streamline care processes and improve overall efficiency. By incorporating ALOS into resource planning and relief program design, policymakers can better strike a delicate balance between meeting immediate staffing needs and controlling operational expenses. This would allow hospitals to maintain quality of care while safeguarding financial sustainability.

This study acknowledges certain limitations that warrant discussion. First, the utilization data obtained were at the hospital level, while the data on COVID-19 cases and deaths were available only at the county level. Nevertheless, county-level cases and deaths can serve as reasonable proxies for assessing the demand for hospitals within each county. Second, it is important to note that this analysis focused exclusively on the state of California. Despite this regional limitation, the study holds significant value as California is the largest and most populous state in the United States, with the highest number of COVID-19 cases and deaths to date. Lastly, it is worth highlighting that this study reflects a period when COVID-19 vaccinations were not yet widely accessible. The introduction of vaccines occurred in the latter part of 2020 Q4, and the phased implementations of vaccines continued throughout 2021 Q1, which was the final quarter considered in this study. Consequently, future investigations into the impact of COVID-19 on hospital utilization should consider examining periods following the widespread adoption of the vaccine. We believe these limitations provide valuable insights into the contextual considerations surrounding the study, warranting further exploration and investigation into the evolving dynamics of COVID-19 and its influence on hospital utilization.

## Conclusions

In this cross-sectional study of 56 California counties, we observed a positive correlation between ALOS and COVID-19 patient ICU visits, as well as a negative correlation with overall COVID-19 patient visits. Additionally, we found a positive association between ALOS and average revenue per patient discharge. These findings were consistent across both rural and urban counties in the State of California. However, the negative association between ALOS and the number of COVID-19 patient visits was observed only within urban counties, whereas no such relationship was apparent in rural counties. The insights into how different types of patient visits influence ALOS can guide health care administrators and policymakers in optimizing resource allocation and staff utilization, especially during periods of high demand or disruptive events.

## Data Availability

County-level COVID-19 data is publicly available and can be obtained from California Open Data Portal (https://data.ca.gov/group/covid-19). Hospital-level utilization data is publicly available and can be obtained from California Health and Human Services Open Data Portal (https://data.chhs.ca.gov).
